# Neurogenic Hypotension and Bradycardia Modulated by Electroacupuncture in Hypothalamic Paraventricular Nucleus

**DOI:** 10.3389/fnins.2022.934752

**Published:** 2022-07-22

**Authors:** Stephanie C. Tjen-A-Looi, Liang-Wu Fu, Zhi-Ling Guo, Yiwei D. Gong, Anh Thi Ngoc Nguyen, Ai-Thuan P. Nguyen, Shaista Malik

**Affiliations:** College of Health Sciences, Susan Samueli Integrative Health Institute, University of California, Irvine, Irvine, CA, United States

**Keywords:** opioids, parasympathetic, CCK, somatosensory, serotonin

## Abstract

Electroacupuncture (EA) stimulates somatic median afferents underlying P5-6 acupoints and modulates parasympathoexcitatory reflex responses through central processing in the brainstem. Although decreases in blood pressure and heart rate by the neural-mediated Bezold-Jarisch reflex responses are modulated by EA through opioid actions in the nucleus tractus solitarius and nucleus ambiguus, the role of the hypothalamus is unclear. The hypothalamic paraventricular nucleus (PVN) is activated by sympathetic afferents and regulates sympathetic outflow and sympathoexcitatory cardiovascular responses. In addition, the PVN is activated by vagal afferents, but little is known about its regulation of cardiopulmonary inhibitory hemodynamic responses. We hypothesized that the PVN participates in the Bezold-Jarisch reflex responses and EA inhibits these cardiopulmonary responses through the PVN opioid system. Rats were anesthetized and ventilated, and their heart rate and blood pressures were monitored. Application of phenylbiguanide every 10 min close to the right atrium induced consistent depressor and bradycardia reflex responses. Unilateral microinjection of the depolarization blockade agent kainic acid or glutamate receptor antagonist kynurenic acid in the PVN reduced these reflex responses. In at least 70% of the rats, 30 min of bilateral EA at P5-6 acupoints reduced the depressor and bradycardia responses for at least 60 min. Blockade of the CCK-1 receptors converted the non-responders into EA-responders. Unilateral PVN-microinjection with naloxone reversed the EA inhibition. Vagal-evoked activity of the PVN cardiovascular neurons was reduced by 30 min EA (P5-6) through opioid receptor activation. These data indicate that PVN processes inhibitory cardiopulmonary reflexes and participates in EA-modulation of the neural-mediated vasodepression and bradycardia.

## Introduction

Vasovagal syncope mediated neurally includes activation of cardiopulmonary afferents and medullary cardiovascular centers (Iwase et al., 2014). Activation of the cardiopulmonary unmyelinated chemosensitive vagal afferents by prostaglandin-E_2_, veratrum alkaloids, capsaicin, serotonin (5-HT), or intravenous phenylbiguanide (PBG) reflexly decreases heart rate and blood pressure (Coleridge et al., 1965; Coleridge and Coleridge, 1980; Jeggo et al., 2005). This phenomenon is described and characterized as the Bezold-Jarisch reflex or cardiopulmonary reflex (Krayer, 1961; Aviado and Guevara, 2001). Several studies have demonstrated that brain stem regions process the Bezold-Jarisch reflex responses. In this regard, the negative chronotropic response following PBG administration results from excitation of cardiac parasympathetic neurons in the nucleus ambiguus (McAllen and Spyer, 1976; Verberne and Guyenet, 1992; Wang et al., 2000; Tjen-A-Looi et al., 2012). A number of other medullary regions, such as the rostral and caudal ventrolateral medulla (rVLM and cVLM) and nucleus tractus solitarius (NTS; Verberne and Guyenet, 1992; Barman et al., 2005; Jeggo et al., 2005; Tjen-A-Looi et al., 2018), process the inhibitory hemodynamic response.

There are many sources of afferent input to the paraventricular nucleus (PVN). The PVN is activated by conditions, such as coronary artery ligation, water-immersion stress, as well as hypertension (Ciriello et al., 1984; Takeda et al., 1991; Zhong et al., 2008). Vagal afferents also innervate the hypothalamus PVN. In this regard, few studies have shown that vagal nerve stimulation also activates neurons in the PVN (Coleridge et al., 1965; Cunningham et al., 2008; Fawley et al., 2021). Although vagal afferent input in PVN is reported, it is unclear if the hypothalamic PVN participates and processes the cardiopulmonary vagal input during the Bezold-Jarisch reflex response.

Electroacupuncture (EA), a form of somatic afferent stimulation with low current and low frequency (2–4 mA, 2 Hz, 0.5 ms), reduces sympathetic over-excitation and elevated blood pressure involving the medulla and hypothalamus region PVN (Tjen-A-Looi et al., 2003, 2007, 2016b; Li et al., 2016). We also have shown that PBG-induced vasodepression and bradycardia can be reduced by EA through actions in the medullary nuclei NTS and nucleus ambiguus (Tjen-A-Looi et al., 2013, 2018). The current study investigates if the hypothalamic region PVN also participates in the EA-inhibition of the reflex responses vasodepression and bradycardia.

Opioidergic nerve fibers, such as enkephalinergic and β-endorphinergic, are present in the PVN (Appel et al., 1986). These fibers are located in close proximity to PVN neurons (Tjen-A-Looi et al., 2016b). In addition, somatic afferent stimulation (EA) induces neuronal c-Fos expression in the PVN and influences PVN neurons in altered physiological conditions, such as stress evoked by immobilization or the maze test (Pan et al., 1994; Kim et al., 2009). Our previous study has shown that EA inhibits elevated sympathoexcitation in cardiovascular PVN neurons and blood pressure reflex responses through opioids in the PVN (Tjen-A-Looi et al., 2016b). The current study determines if the PVN-opioid system also participates during EA’s inhibition of vasodepressive and bradycardia reflex responses. We hypothesized that the PVN participates in the Bezold-Jarisch responses and EA modulation of parasympathoexcitatory cardiovascular responses through an opioid mechanism. This work has been published in preliminary form (Tjen-A-Looi et al., 2015; 2016a).

We have shown that EA responsiveness is observed in the majority of subjects, while non-responders to EA count for 25–30% (Li et al., 2013). The activity of cholecystokinin-1 (CCK-1) receptor appears to interfere with the effectiveness of EA-inhibition on sympathoexcitatory reflex response in the rVLM in about 25–30% of subjects (Li et al., 2013). In this regard, we investigate the role of the CCK system in PVN during the parasympathoexcitatory cardiopulmonary reflex responses in rats not responsive to EA (P5-6) modulation of the Bezold-Jarisch reflex.

## Materials and Methods

### Surgical Procedures

Experimental preparations and protocols, AUP 19-112, were approved by the Institutional Animal Care and Use Committee of the University of California, Irvine. The study conformed to the American Physiological Society’s “Guiding Principles for Research Involving Animals and Human Beings” (World Medical Association, and American Physiological Society, 2002). Studies were performed on 62 adult Sprague–Dawley male rats (400–650 g). Anesthesia was induced with ketamine (100 mg/kg, im) and xylazine (10 mg/kg, im). Additional doses of xylazine (0.02 mg/kg, iv) were given as necessary to maintain an adequate level of anesthesia, which is determined by the lack of response to noxious toe pinch, a respiratory pattern that followed the respirator, and a stable blood pressure and heart rate. The trachea was exposed and intubated to ventilate animals with a respirator (Model 683, Harvard Apparatus, Holliston, MA, United States). A carotid artery was cannulated and attached to a pressure transducer (P23XL, Ohmeda, Laurel, MD, United States) to monitor blood pressure, while the heart rate was derived from the pulsatile blood pressure signal. Arterial blood gases and pH were measured periodically with a blood gas analyzer (ABL5, Radiometer America, Brea, CA, United States) and maintained in the normal physiological status (pH, between 7.35 and 7.45) with 8% sodium bicarbonate. Body temperature monitored with a rectal thermistor (Model 44TD) was maintained between 36 and 38°C with a heat pad and a lamp. The right jugular vein was cannulated with the tip of the cannula placed close to the right ventricle of the heart for administration of PBG. The femoral vein was cannulated for the delivery of fluids. The cervical vagus was isolated and stimulated with a bipolar stimulating electrode connected to an isolation unit and a stimulator (Grass, Model S88) using low current (0.7–1 mA, 10 Hz and 0.5 ms) to elicit a decrease in the heart rate. The stimulating electrode was held in place with non-neurotoxic dental impression material (Pentron, Wallingford, CT, United States). Afterward, the cervical incision was closed to prevent desiccation.

The head of the animal was stabilized with a Kopf stereotaxic head frame to facilitate craniotomy and partial removal of the dura to access the cortex. Recording a pipette and a microinjection probe was positioned perpendicularly to the dorsal surface of the cortex with visual approximation of 0.8–1.5 mm lateral to the midline and 1.7–2.0 mm caudal to the bregma using coordinates taken from the atlas of Paxinos and Watson (2009). The microinjection probe [a modified CMA microdialysis AB probe that was 14-mm long with a tip diameter of 0.24 mm (CMA, Stockholm, Sweden)] that lacked the microdialysis membrane (Guo et al., 2009; Tjen-A-Looi et al., 2011) was advanced ventrally 7.2–7.6 mm to reach the PVN. Microinjection of DLH confirmed the cardiovascular region of the PVN with a small increase in blood pressure (8–10 mmHg). Unilateral insertion of the probe allowed a more optimal physiological condition than bilateral insertions. The probe was connected to a CMA 402 syringe pump (CMA, Stockholm, Sweden) to deliver 50 nl at a rate of 0.3 μl/min over a 10-s period 2 min prior to next PBG application. The extracellular recording electrode was a 3-barrel glass pipette used to evaluate PVN neuronal activity and iontophoresis of drugs. One barrel of the pipette was filled with either saline or naloxone. The other two barrels contained a platinum recording electrode in 0.5-M sodium acetate, containing 2% Chicago blue dye (Sigma Chemical, St. Louis, MO, United States) or a current-balancing solution of 4-M NaCl. The recording pipette was lowered to a depth of 7 mm and advanced at increments of 1–2 μm to locate, identify, and characterize cardiovascular PVN neurons (see below).

### Drugs

Hypotension and bradycardia were induced with PBG (4–10 μg/ml/kg, iv) administration every 10 min. The participation of the PVN and the role of glutamatergic system during the Bezold-Jarisch reflex response was examined, respectively, with microinjection of the depolarizing agent kainic acid (KA, 1 mM, 50 nl) (Talman et al., 1981; Tjen-A-Looi et al., 2011) and the non-selective glutamate antagonist kynurenic acid (Kyn, 50 mM, 50 nl) (Zhou et al., 2006). The role of opioids in PVN during effects of EA was evaluated by microinjection (hemodynamic studies) and iontophoresis (electrophysiological studies) of the non-specific opioid antagonist naloxone (100 nM, 50 nl, 2 min at 120 nA, Sigma-Aldrich, St. Louis, MO, United States) (Tjen-A-Looi et al., 2007) either right or left into the PVN (Ray et al., 1991; Chao et al., 1999; Crisostomo et al., 2005; Tjen-A-Looi et al., 2007) 5 min after the end of EA. The influence of CCK-8 agonist (0.2 mM, American Peptide Company, Sunnyvale, CA, United States) (Heinricher et al., 2001) and CCK-1 receptor antagonist devazepide (alternative names: MK-329 or L-364718, 0.5 mM, Sigma-Aldrich, St. Louis, MO, United States) (Crawley, 1992) was examined with and without the effects of EA. Devazepide powder (25 mg) was rinsed in 500-μl ethanol, dissolved in the same amount of polyethylene glycol 400 (Sigma-Aldrich, St. Louis, MO, United States) to achieve a concentration of 25 mg/ml and stored at 4°C. On the day of the experiment, we mixed 1.6 μl of the devazepide solution with 1.6 μl Tocrisolve 100 (Tocris, Ellisville, MS, United States) to achieve a concentration of 12.5 mg/ml that was diluted further with saline to yield a final concentration of 0.2 mg/ml (0.5 mM). The vehicle saline (50-nl microinjection) was used as the control for both CCK-8 and naloxone. Iontophoresis of saline for 2 min served as the control determining PVN neuronal activity. The solution (0.4% ethanol, 0.4% polyethylene glycol 400, and 0.8% Tocrisolve 100 in saline) used to dissolve devazepide functioned as the vehicle control. The timing of delivery of the antagonist and the agonist allowed the observation of acupuncture’s long-lasting effect.

### Stimulating Methods

Repeated stimulation (occurring every 10 min) of cardiopulmonary 5-HT_3_ receptors with PBG or electrical stimulation of vagal afferents (2 Hz, 0.4–0.6 mA, 0.5 ms for 30 s) during electrophysiological recordings was used to induce consistent decreases in blood pressure and heart rate or increases in PVN neuronal activity, respectively. Acupuncture needles (32-gauge stainless steel) were placed bilaterally at the P5-6 acupoints overlying the median nerves located above the paw (Crisostomo et al., 2005). The needles were inserted at P5-6 perpendicularly to a depth of 2–3 mm and were connected to a constant current stimulator with a stimulus isolation unit and Grass stimulator (Model S88, West Warwick, RI, United States). Each set of electrodes was stimulated separately to prevent current flow from one acupoint location to the contralateral side. Correct placement of the needles at the acupoints P5-6 was accessed by observing slight repetitive flexor paw twitches, i.e., a motor threshold, which, periodically, was confirmed during 30-min EA stimulation. The paw twitches were important observations, which confirm stimulation of motor fibers in the median nerve and indicate stimulation of the correct nerve (Li et al., 1998, 2002; Chao et al., 1999). Gallamine triethiodide (4 mg/kg) was administered intravenously after proper placement of acupuncture needles and before application of 30-min EA (2–4 Hz, 1–4 mA, 0.5 ms) to avoid muscle movement during stimulation of the median nerves in studies examining PVN neuronal activity. Of note, motor nerve stimulation does not contribute to the EA inhibition of cardiovascular reflex responses, as EA modulation of reflex cardiovascular responses occurs during muscle paralysis (Li et al., 2006; Tjen-A-Looi et al., 2012). Throughout the EA study of 100–120 min, application of PBG or stimulation of parasympathetic afferents occurred every 10 min superimposed with 30-min EA.

### Paraventricular Nucleus Extracellular Recording

Single-unit activity of PVN neurons was recorded with a platinum electrode inserted into a three-barrel pipette positioned in the PVN. Action potentials were amplified with a preamplifier (Grass P511), attached to a high-impedance probe (Grass H1P5), and then filtered (0.3–10 kHz) and monitored with an oscilloscope (Tektronix 2201). Data were analyzed offline with a computer and CED Spike 2 Windows software. Action potentials were analyzed both visually and using wave shape recognition algorithms to allow detection of similar wave shapes, heights, and latencies of response (Li et al., 2009, 2010). Peristimulus time histograms were constructed for each neuron to assess evoked responses to stimulation of the vagal or median nerves. To assess the evoked responses to stimulation of vagal and median nerves, peristimulus time histograms were constructed for each neuron.

### Identification of Paraventricular Nucleus Neurons

We identified cardiovascular PVN neurons with input from vagal and median nerves, as well as responsiveness to EA using the following criteria. Using peristimulus histograms, PVN neurons were examined for convergence (during a 30-s period of stimulation) of the cervical vagus nerve and median nerves (by brief stimulation at acupoints P5-P6) (Tjen-A-Looi et al., 2012). Peristimulus histograms were constructed with stimuli applied at a frequency of 2 Hz. Then, the convergence was determined with the evoked neuronal activity over a 15-s period to construct peristimulus time histograms with 30 stimuli (Tjen-A-Looi et al., 2003, 2012). To determine cardiac rhythmicity, the relationship between blood pressure and spontaneous PVN neuronal activity (5-min baseline recording) was assessed by both time and frequency domain analyses using arterial pulse-triggered averaging and coherence analysis (Tjen-A-Looi et al., 2007; Li et al., 2009; Moazzami et al., 2010). To further characterize the cardiovascular responsiveness of these PVN neurons, their response to altered baroreceptor input was evaluated following intravenous administration of nitroglycerin (2.5 mg/ml) or phenylephrine (2 mg/ml). Each neuron displaying consistent vagal-evoked activity was examined for EA inhibition (EA responsiveness). Each neuron in the time control protocol was stimulated with a minimum of 10-min EA at P5-6. All other neurons were stimulated with 30-min EA.

## Experimental Protocols

### Hemodynamic Reflex Responses and Electroacupuncture

Hypotension and bradycardia reflex responses were induced by PBG administration every 10 min. The role of PVN during the cardiopulmonary reflex responses was determined with PVN microinjection of KA, as well as with Kyn during the Bezold-Jarisch reflex responses. The mean arterial pressure (MAP) and heart rate reflex responses were repeated 10–11 times to examine the actions of EA. The maximal reflex responses were calculated as the difference in MAP and heart rate pre-PBG administration and at the peak of the cardiopulmonary responses. Following two consistent reflex responses, 30-min EA was applied during three additional PBG administrations, and then followed by PVN microinjection of saline or naloxone to determine the role of opioids during the effects of EA in the course of five or six additional Bezold-Jarisch responses. In another group of rats, the agonist CCK-8 was microinjected in the PVN at the end of 30-min EA to examine disinhibition of the EA effects on hypotension and bradycardia. Baselines of blood pressure and heart rate were recorded and analyzed for consistency to enable evaluations of the reflex responses.

In the rats that were not displaying EA inhibition of the Bezold-Jarisch reflex response, the role of CCK-1 receptor in the PVN was examined. The rats that did not show a reduced PBG-induced cardiopulmonary response during and after 30 min of EA (P5-6) received PVN microinjection of CCK-1 receptor antagonist devazepide or vehicle control. Another 30-min EA treatment was applied after a consistent reflex response to determine the conversion of the EA poor-responder to the EA responder. The long-lasting EA effects were observed with 4–6 additional reflex responses. Saline or naloxone was microinjected into the PVN 5 min after the second EA.

### Paraventricular Nucleus Neuronal Activity During Electroacupuncture and Opioid Receptor Blockade

Each PVN neuron that was studied received three types of convergence. Neurons were evaluated for input from median nerves by activation of acupoints P5-6, stimulation of cervical vagal (afferent) nerves, and responsive to baroreceptor challenge. The neurons were analyzed to display cardiac rhythmicity, and the responsiveness to EA was determined by a significant reduction of the repeated vagal-evoked responses. In the time control protocol either before or after 10 repeated PVN vagal-evoked activity, the neurons were examined for EA responsiveness with a minimum of 10- to 20-min EA stimulation at P5-6. In the EA protocols, repeated PVN vagal-evoked neuronal activity was recorded every 10 min, while EA was delivered continuously for 30 min. The effect of EA was evaluated following two consistent vagal-evoked neuronal activity. At the end of EA, saline or naloxone was iontophoresed onto the PVN neurons. Thereafter, we evaluated the prolonged EA inhibition of the neuronal activity observed after 30-min EA. The subgroup of the characterized neurons also was tested for evoked activity to PBG administration.

### Histology

At the end of each experiment, animals were euthanized under deep α-chloralose anesthesia followed by injection of saturated KCl. Recording and/or microinjection sites were marked by either iontophoresis and/or microinjection of 2% Chicago blue dye. Thereafter, the brain was removed and fixed in 10% paraformaldehyde for at least 2 days. Brains were sliced with a microtome cryostat (Leica CM 1850) at 40-μm coronal sections. Recording and microinjection sites were reconstructed from the dye spots with the aid of a microscope (Nikon) and software (Corel presentation). The sites were plotted on coronal sections separated by a 0.3-mm interval.

## Data Analysis

Data are presented as means ± SEM. Evoked-PVN activity was measured as the increase in number of spikes above the baseline. Changes in MAP and heart rate are presented as bar histograms. The increase in cellular activity and decreases in blood pressure and heart rate before and after delivery of experimental drugs or saline were compared by a one-way ANOVA followed by *post hoc* with the Student–Newman–Keuls test. Data were analyzed with the Kolmogorov–Smirnov test for normal data distribution and normalized if needed with Friedman’s repeated measures ANOVA on Ranks and a Dunnett’s *post hoc* test. All data analyses were performed with Sigma plot and Sigma Stat (Jandel Scientific, San Jose, CA, United States). The 0.05 probability level was used to detect significant differences.

We also evaluated time and frequency relationships between PVN neuronal activity and arterial blood pressure using pulse-triggered averaging, as well as coherence analysis. Time domain analyses involved arterial pulse-triggered averaging. A threshold was set at the systolic phase of the arterial pulse, and spike height discrimination and waveform recognition were used to sort action potentials during the evaluation period of 300 s. Time domain analysis averages of the arterial pulse and histograms of PVN neuronal activity were constructed as displayed in our previous studies (Shin et al., 1995; Li et al., 2010). Frequency domain analysis involved assessment of the coherence between PVN neuronal activity and arterial blood pressure using a Fast Fourier Transform (FFT) algorithm. We recorded data using a sampling rate of 10,000 Hz. Reconstructed data utilized every tenth sample, including assessment of the mean and peak amplitudes and the maximum and minimum slopes of the original PVN spike to preserve the action potentials. The spikes were sorted and identified with a window discriminator to construct bar histograms prior to coherence analysis. The number of data sections (15–20 each lasting for 12.8 s) was chosen to determine the average histogram. The autospectra of PVN discharge and arterial blood pressure were generated with FFT algorithm. Thus, coherence was generated with seven overlapping windows, each with a length of 12.8 s, consisting of 256 bins, with bin widths of 50 ms. The auto-spectral analysis was adopted from Shin et al. (1995) using contiguous segments of 256 beats with 50% overlap between the segments. The frequency resolution was 1/12 s or 0.08 Hz. The coherence function (normalized cross-spectrum) provided a measure of the strength of linear correlation of PVN neuronal activity and blood pressure at each frequency. Coherence values of ≥0.5 were chosen to reflect a statistically significant relationship between the PVN spikes and arterial blood pressure (Tjen-A-Looi et al., 2007).

## Results

### Role of Paraventricular Nucleus in Cardiopulmonary Reflex Responses

**FIGURE 1:**
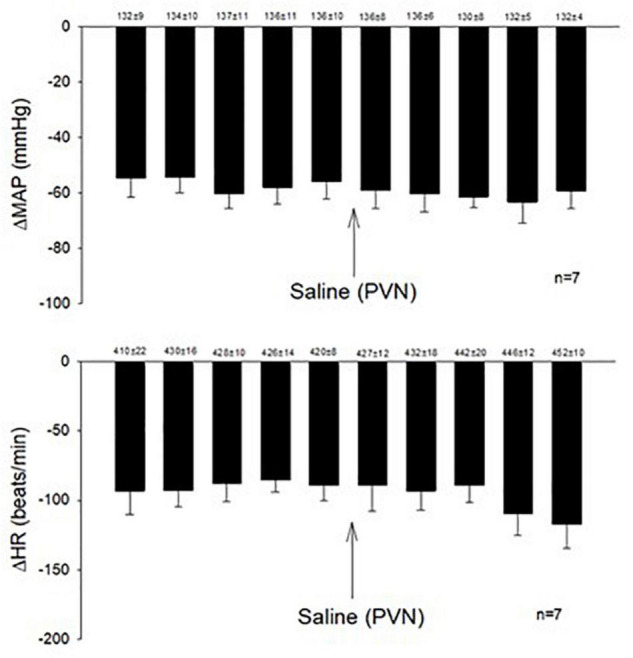
Repeated administration of PBG induced consistent Bezold-Jarisch reflex responses. The depressors and bradycardia reflex responses were not altered after microinjection of saline into the PVN. Baselines blood pressures and heart rates in mean ± SEM shown above bars were not significantly different throughout experiments.

Microinjection of KA (transient depolarization blockade, *n* = 4) and Kyn (the antagonist of glutamate receptors, *n* = 4) into the PVN (0.6–1.9 mm lateral, 1.6–2.1 mm caudal, 7.2–7.6 mm ventral) significantly reduced (*p* < 0.05) the Bezold-Jarisch responses in rats (Table 1). The baseline levels of blood pressure and heart rate were not altered following microinjection of KA or Kyn. Repeated PBG administration every 10 min showed consistent decreases in blood pressure and heart rate before and after microinjection of saline. The baselines (displayed above each bar) were not affected significantly throughout the experiment (Figure 1).

**TABLE 1:** The role of PVN and glutamatergic system in PVN during Bezold-Jarisch reflex responses.

	Kainic acid (KA) – PVN microinjection				Kynurenic acid (Kyn) – PVN microinjection			
	Pre	Post			Pre	Post		
	5 min	5 min	20 min	30 min	5 min	5 min	20 min	30 min
ΔMAP	−51.7 ± 7.3	−29.2 ± 7.4*	−24.0 ± 10.1*	−44.2 ± 6.8	−40.0 ± 8.7	−19.0 ± 3.4*	−27.7 ± 7.7	−38.0 ± 8.4
ΔHR	−115.0 ± 24.3	−54.5 ± 10.2*	−42.0 ± 20.5*	−73.7 ± 15.9	−71.2 ± 10.7	−22.5 ± 4.8*	−63.0 ± 27.4	−76.2 ± 29.4

Values are expressed as mean ± SEM. *Significantly different compared with pre-microinjection [p < 0.05, post-KA vs. pre-KA (n = 4) and post-Kyn vs. pre-Kyn (n = 4)].

*ΔMAP, change in mean arterial pressure; ΔHR, change in heart rate.*

### Effects of Electroacupuncture on Phenylbiguanide-Evoked Cardiopulmonary Reflex Responses

**FIGURE 2:**
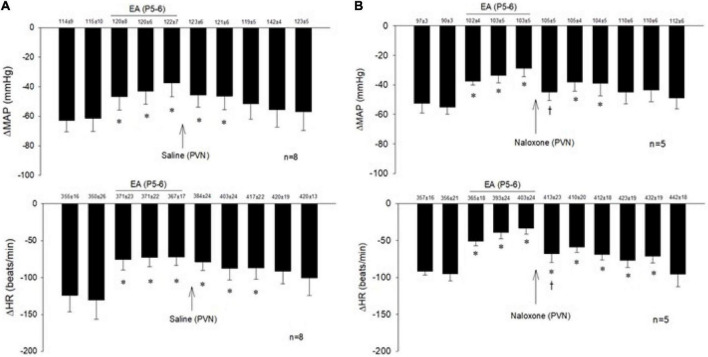
Electroacupuncture (EA) at P5-6 acupoints reduced the Bezold-Jarisch responses evoked by PBG every 10 min. EA reduced the inhibitory cardiovascular responses for at least 60 min **(A,B)**. Blockade of opioid receptors with naloxone in the PVN reversed the inhibitory effects of EA on cardiopulmonary reflex responses **(B)** in contrast to saline **(A)**. Baselines blood pressures and heart rates in mean ± SEM shown above bars were not significantly different throughout experiments. *Significant difference compared with control PBG response. ^†^Significant difference from the preceding PBG response.

The consistent decreases in blood pressure and heart rate with repeated stimulation of the cardiopulmonary 5-HT_3_ receptors with PBG were examined before, during, and after EA at P5-6 overlying the median nerve. The PBG-evoked cardiopulmonary responses were reduced during and after application of 30-min EA. The modulatory effect of EA lasted 50–80 min. Saline microinjection into PVN did not affect the EA inhibition of the cardiopulmonary responses (Figure 2A). In contrast, naloxone, the opioid receptor blocker, reversed the EA effect on blood pressure and heart rate in a separate group of rats (Figure 2B). Baselines of blood pressure and heart rate before each reflex response were consistent throughout the experiments.

### Cholecystokinin System in Paraventricular Nucleus During Electroacupuncture Inhibition of the Bezold-Jarisch Reflex Responses

**FIGURE 3:**
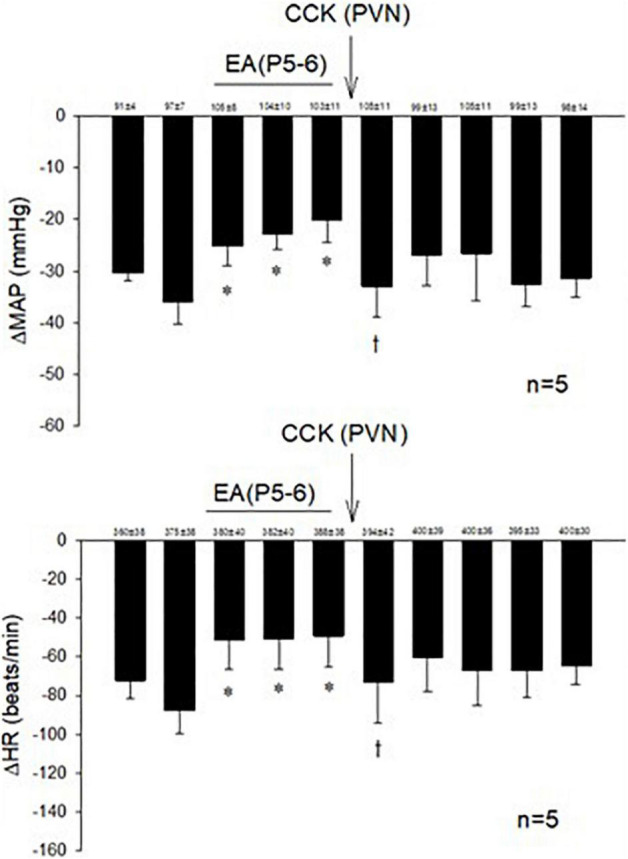
The role of CCK in the effects of EA during hypotensive and bradycardia responses. PVN microinjection of the agonist CCK-8 inhibited the effect of EA modulation of the cardiopulmonary responses. Baselines blood pressures and heart rates in mean ± SEM shown above bars were not significantly different throughout experiments. *Significant difference compared with control PBG response. ^†^Significantly different compared with the preceding PBG response.

Examining the role of the CCK system in the PVN during actions of EA, we observed that microinjection of the CCK-8 agonist in the PVN inhibited the effect of EA in responsive rats (Figure 3).

### Conversion of Non-responders Into Responders by Blockade of Cholecystokinin Receptors

**FIGURE 4:**
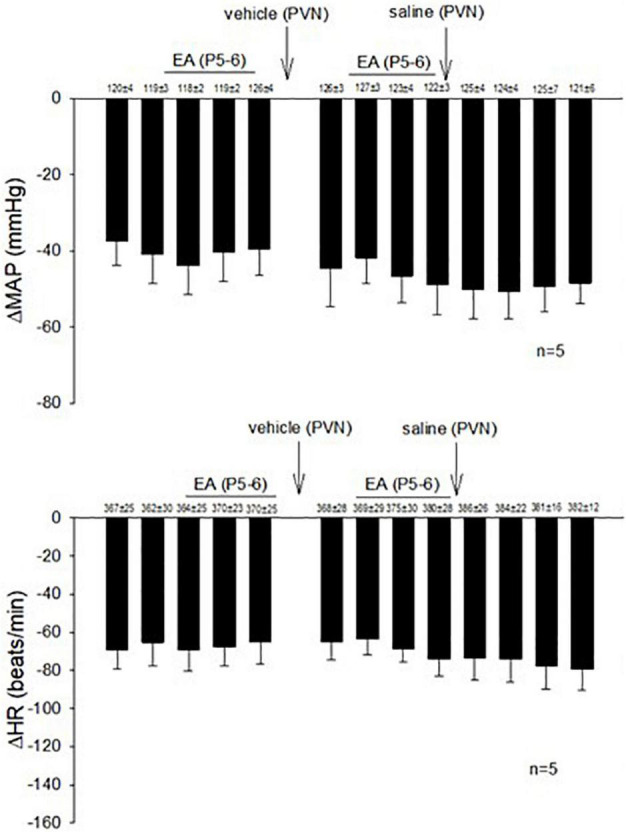
Rats non-responsive to EA at the P5-P6 acupoint (overlying the median nerve) were tested with second EA treatment to modulate the PBG-induced Bezold-Jarisch reflex responses. Vehicle control and saline control do not affect the lack of responsiveness to EA in the non-responders.

**FIGURE 5:**
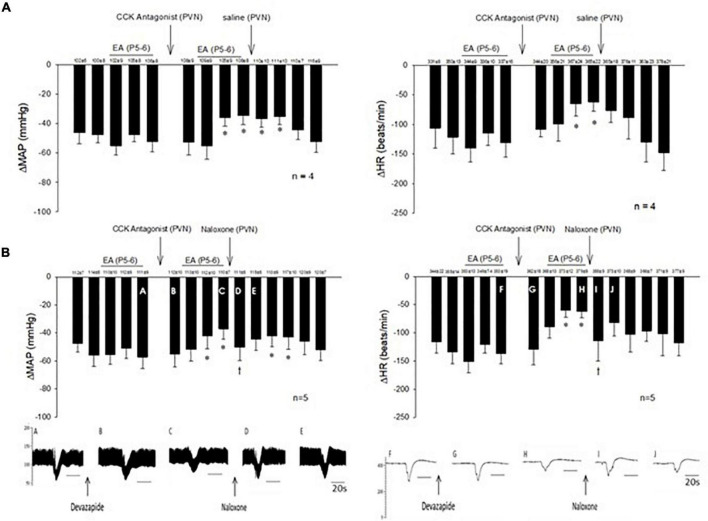
Non-responders to EA on the Bezold-Jarisch responses were converted into responders following blockade of the CCK_1_ receptor in the PVN. Unlike the responders, EA inhibition of the depressor and bradycardia was not observed in the non-responders. However, repeat EA reduced the depressor and bradycardia after microinjection of devazepide (the CCK antagonist) into the PVN in the non-responder **(A)**. The effectiveness of EA, after CCK1 blockade, was reversed with naloxone **(B)**. An individual set of data on blood pressure (A–E) and heart rate (F–J) tracings is displayed below the bar histograms. The letters above the tracings correspond with the letters shown in the bars. *p < 0.05, indicates significant reduction in heart rate and blood pressure during effects of EA compared with PBG responses before EA. ^†^*p* < 0.05, indicates significant reversal of EA effect following naloxone compared with PBG response prior to blockade.

In 14 non-responsive rats, we observed absence of EA modulation of the Bezold-Jarisch responses in contrast to 48 EA-responder rats. To confirm non-responsiveness to EA, a second EA application was delivered following 30 min of the resting period (Figure 4). In another group of the non-responder rats, we observed that blockade of the CCK-1 receptor with devazepide in the PVN converted a non-responder into a responder rat to EA (P5-6) modulation of the cardiopulmonary reflex responses (Figure 5A) in contrast to vehicle and saline microinjections (Figure 4). On the other hand, we observed reversal of the decreased reflex response by EA following naloxone microinjection in the converted rats (Figure 5B, Panels D,I).

### Profile of Paraventricular Nucleus Neuronal Activity

**FIGURE 6:**
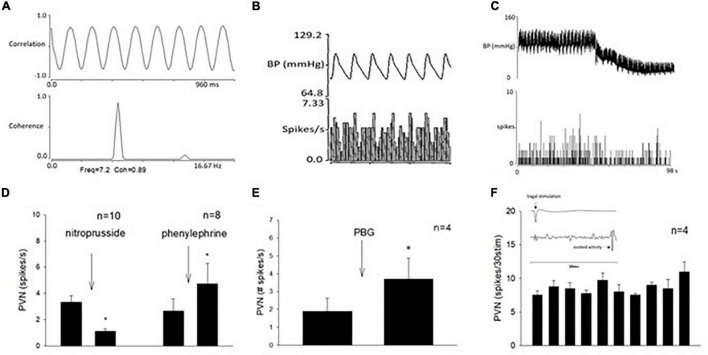
Methods used to classify paraventricular nucleus (PVN) neurons that received input from the vagal nerve, the median nerve, and the baroreceptor. **(A)** Displays strong correlation and coherence between PVN neuronal activity and blood pressure. **(B)** Shows time domain analysis of a strong relationship between PVN discharge and blood pressure. **(C)** Displays recording of blood pressure (BP) and activity of PVN neuron (receiving vagal input) after intravenous nitroprusside. Group data of PVN neuronal activity following administration of nitroprusside and phenylephrine **(D)**. **(E)** Shows with a bar histogram the evoked activity in the PVN following PBG intravenous administration. **(F)** Displays consistent evoked PVN activity by repeated cervical vagal nerve stimulation. The inlay displays time of vagal stimulation and its evoked spike in PVN **(F)**. *Indicates significant difference in PVN activity following administration of nitroprusside or phenylephrine **(D)** or PBG **(E)**.

**FIGURE 7:**
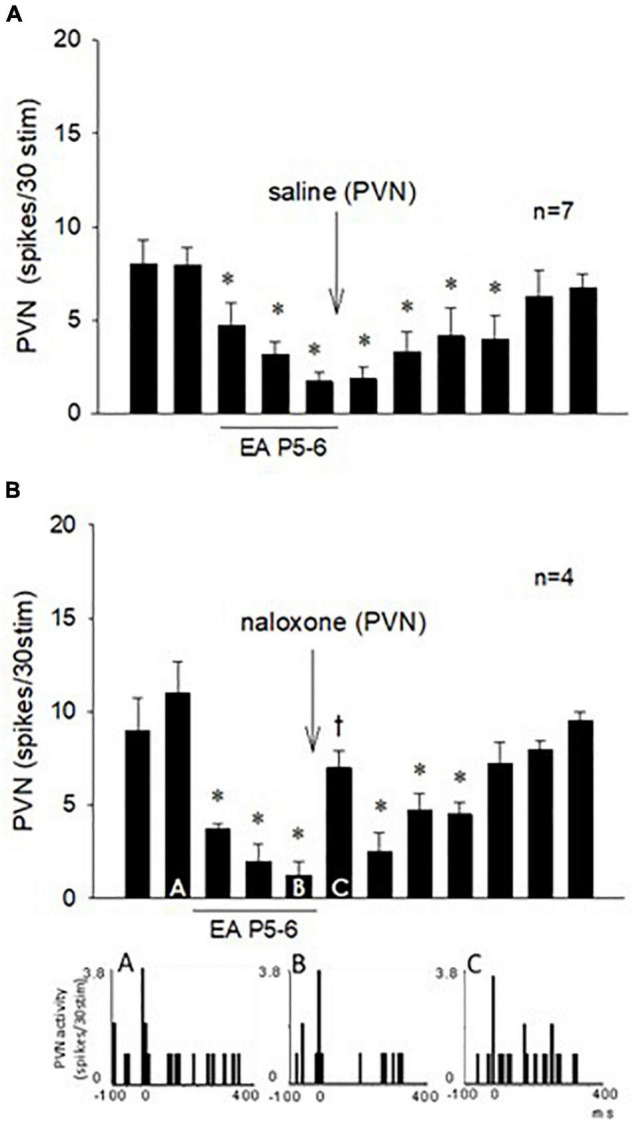
Repeated cervical vagus nerve stimulation evoked PVN activity that was reduced by EA for at least 70 min. Saline microinjection did not influence the EA inhibition **(A)**. In contrast, blockade of opioid receptors in the PVN reversed the EA action (Bar **C**) followed by the long-lasting EA effect for another 30 min **(B)**. Peristimulus histograms **(A–C)** display discharge activity of a cardiovascular PVN neuron with input from vagal, baroreceptor, and EA-activated median nerves. The letters A, B, and C in the peristimulus histograms correspond with the letters in the bars. *Significant difference compared with control PBG response. ^†^Significant difference from the preceding vagal-evoked activity.

In addition to cardiopulmonary reflex responses, we examined the neuronal discharge of cardiovascular neurons in the PVN. Activity of neurons in the PVN were identified and characterized prior to their responses to repeated stimulation of the vagal nerve and 30-min EA. We recorded 41 cells and characterized 19 neurons with the vagal nerve, as well as the median nerve (15-s stimulation at P5-6) convergence. Fifteen of these neurons also were responsive to EA. All these 15 cells were responsive to unloading and loading of baroreceptors (either nitroprusside, or phenylephrine, or both) and displayed cardiac rhythmicity and strong coherence and correlation between PVN discharge and arterial blood pressure (Figures 6, 7). Activity of four neurons examined for PBG-evoked input was increased from 1.9 ± 0.7 to 3.7 ± 1.1 (spikes/s) (Figure 6E), while the blood pressure decreased from 112.7 ± 7.7 to 77.3 ± 17.3 (mmHg) and the heart rate from 357.3 ± 16.3 to 226.6 ± 22.1 (beats/min). We observed consistent evoked PVN neuronal activity (*p* = 0.21), ranging from 7.6 ± 0.5 to 11. ± 1.4 (spikes/30 stimuli) in four neurons (Figure 6F), indicating the viability of these PVN neurons to examine actions of EA.

### Paraventricular Nucleus Neuronal Response to Electroacupuncture and the Role of Opioid

The effect of EA on the repeated vagal nerve-evoked PVN neuronal activity was observed in the other 11 neurons (Figure 7). In seven PVN neurons, we observed that 30-min EA reduced the evoked activity for at least 70 min during and after acupuncture, while saline iontophoresis did not influence the EA inhibition (Figure 7A). We observed in four other PVN neurons that blockade of the opioid receptors with naloxone reversed the EA-inhibitory action on the PVN neuronal activity evoked by stimulation of the vagus nerve (Figure 7B). The evoked activity of an individual PVN neuron analyzed with peristimulus histograms was decreased by EA (while the baseline activity is not changed, Figure 7B, Panels A,B) but increased following iontophoresis of naloxone (Figure 7B, Panel C).

### Histology

**FIGURE 8:**
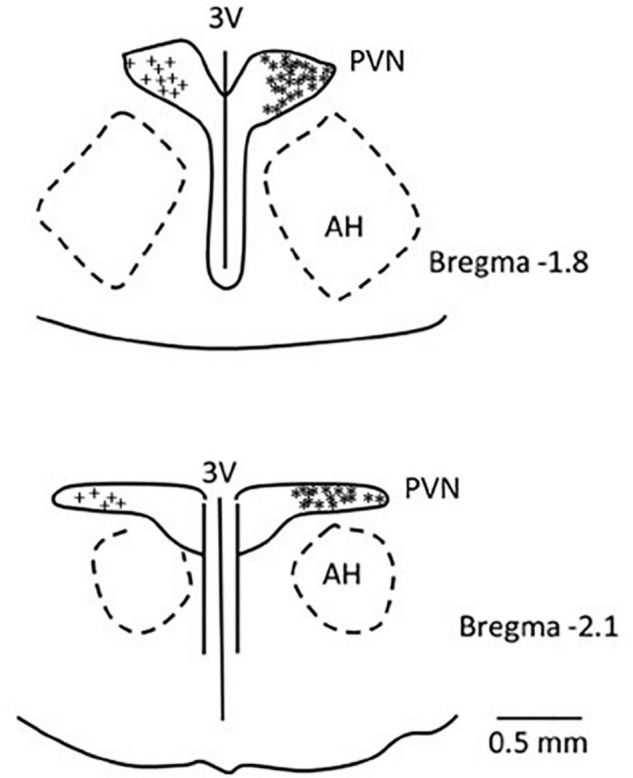
A composite map displays sites of microinjection and recording in the PVN in rats. For ease of presentation, extracellular recording sites in the PVN are displayed on the left (+) and microinjections sites (*) on the right. PVN, paraventricular nucleus; AH, anterior hypothalamic nucleus; 3V, third ventricle.

We confirmed histologically that the sites of microinjection and recording were located within the PVN in accordance with the Paxinos and Watson’s Rat Brain Atlas (Paxinos and Watson, 2009). We observed microelectrode tracks and location of dye injections in the region PVN that is known to contain the neurons related to cardiovascular regulation. A composite map shows the microinjection (*), recording, and iontophoresis (+) sites that are found to be located in the PVN. Sites outside of the PVN were observed to be too lateral and ventral to the PVN (*n* = 4) and were not included in the study (Figure 8).

## Discussion

### Current Findings

We have demonstrated the role of hypothalamic PVN during the PBG-induced Bezold-Jarisch reflex responses, as well as reduction of these responses by EA through PVN opioid receptor activation. We observed that activation of the cardiopulmonary 5-HT_3_ receptor with PBG evoked significant decreases in blood pressure and heart rate through the PVN glutamatergic system. These profound hemodynamic decreases were minimized by 30-min EA applied bilaterally at P5-6 acupoints through opioids in the PVN for a prolonged period of time. In addition, we observed that the PVN cardiovascular neurons processed the action of EA inhibition on parasympathetic or cardiopulmonary input through the opioid system. In this regard, blockade of the opioid receptor in the PVN reversed the action of EA on blood pressure, heart rate, and vagally evoked activity of PVN cardiovascular neurons. About 30% of the animals did not respond to the EA application in reducing the cardiopulmonary reflex responses defined as non-responders. In parallel with the sympathoexcitatory studies in rVLM (Li et al., 2013), the current study showed that blockade of the CCK-1 receptor converted the non-responder to EA into EA-responder animals. The PVN opioid system also contributed to the EA effects on these converted rats.

### Phenylbiguanide-Induced Cardiopulmonary Reflex Responses

Several studies have demonstrated that the Bezold-Jarisch or cardiopulmonary reflex responses induced by activation of 5-HT_3_ receptors are processed in the brainstem. Furthermore, the sympathoinhibitory component of the cardiopulmonary reflex response shares a pathway similar to the activity of the baroreceptor (Verberne and Guyenet, 1992). Specifically, the medullary regions, such as the NTS, rVLM, cVLM, and nucleus ambiguus (Verberne and Guyenet, 1992; Jeggo et al., 2005; Tjen-A-Looi et al., 2011, 2014), process the baroreceptor-related inhibitory input, leading to reversal of inhibitory hemodynamic responses. In the same regions, the bradycardia in response to PBG stimulation by excitation of cardiac parasympathetic neurons is processed, in part, through the nucleus ambiguus (Tjen-A-Looi et al., 2012). On the other hand, the decreased blood pressure response is evoked by activation of the cardiovascular neurons in the NTS and cVLM that, in turn, inhibit rVLM activity (Verberne and Guyenet, 1992).

**FIGURE 9:**
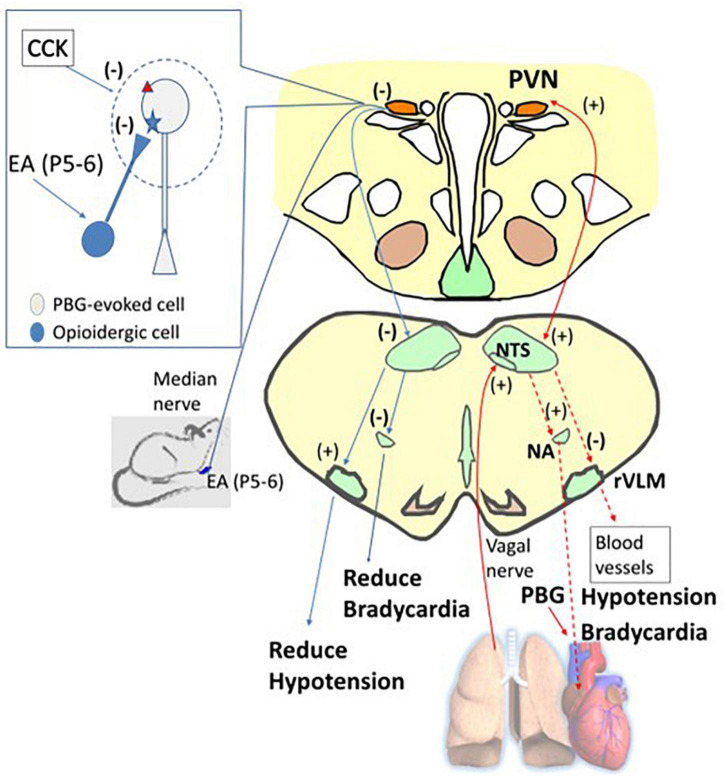
A diagram displays the afferent (the solid red arrow) and efferent (the dashed red arrow) nerves and projections involved in phenylbiguanide (PBG) induced cardiopulmonary reflex response modulated by electroacupuncture (EA). For ease of display, on the right is the PBG-related neuronal pathway (red arrows), while on the left is the reversal of the evoked activities, leading to reduction of hypotension and bradycardia by EA inhibition (blue arrows). The dialogue box on the left shows opioid and CCK systems observed in non-responders. The opioid receptor is shown as a blue star and the CCK receptor as a red triangle. PVN, paraventricular nucleus; NTS, nucleus tractus solitaries; rVLM, rostral ventrolateral medulla; NA, nucleus ambiguus. (+), excitatory projection. (–), inhibitory projection. NTS-rVLM, inhibitory projection through GABAergic cVLM neurons. NA-Heart, inhibitory projection through acetylcholinergic neurotransmission.

In the hypothalamus, vagal nerve stimulation has been shown to activate neurons in the PVN (Cunningham et al., 2008; Bassi et al., 2017). The PVN projects to the NTS that eventually activates the nucleus ambiguus regulating the heart rate through parasympathetic efferents and reduces rVLM activity, sympathetic outflow, and cardiovascular responses (Luiten et al., 1985; Verberne and Guyenet, 1992; Tjen-A-Looi et al., 2012). The present study shows the involvement of the glutamatergic system in the PVN during the Bezold-Jarisch response. In this regard, our current study demonstrates parasympathoexcitatory cardiopulmonary input in the PVN, leading to decreases in blood pressure and heart rate possibly employing the PVN-NTS pathway shown to be involved in cardiorespiratory responses to acute hypoxia (Ruyle et al., 2019). The neurons that receive vagal input and also respond to baroreceptor activity highly likely involve the PVN-NTS neuronal pathway. Collectively, the PBG-induced cardiopulmonary reflex responses include the hypothalamic PVN and may share a common pathway related to the arterial baroreflex (Figure 9).

### Modulation of the Autonomic Nervous System by Electroacupuncture

We have shown that stimulation at specific acupoints activating underlying somatic nerves during application of EA modulates autonomic responses to reduce not only elevated sympathetic tone but also evoked parasympathetic activity (Tjen-A-Looi et al., 2004, 2018). Our studies have shown an interaction between specific somatosensory activity and parasympathoexcitatory reflex responses. We have shown that, through the medulla, hypotension and bradycardia induced by gastric distention during hypercapnia or activation of cardiopulmonary 5-HT_3_ receptors are modified with 30-min activation of somatosensory nerves by EA overlying the median nerves or deep peroneal nerves through neurochemicals, such as γ-aminobutyric acid (Tjen-A-Looi et al., 2013, 2014, 2018).

The present data show the role of the opioid system in the PVN during EA inhibition of both hypotension and bradycardia responses. Blockade of the opioid receptors reverses the effects of EA on the hemodynamic responses and the vagally evoked PVN neuronal activity. We have observed that opioid receptor blockade in the PVN does not affect the baseline blood pressure (Tjen-A-Looi et al., 2016b), suggesting that the opioid system in the PVN does not influence basal tone. Future studies are warranted to investigate the role of opioid receptor subtypes during the actions of EA on PBG-induced neural-mediated hypotension and bradycardia responses in the PVN.

### Non-responders

A number of preclinical studies report that CCK neurotransmitter system is associated with responsiveness and poor responsiveness to acupuncture treatment (Kim et al., 2017). Conversion of non-responders into acupuncture-induced analgesia responders has been shown with an anti-CCK antibody or antisense CCK-8 injected into intracerebroventricular or intrathecal space (Han et al., 1985). Concurrently, we have shown in the present study that activation of CCK receptors in the PVN with the CCK-8 agonist reverses the effects of EA on the neurogenic-mediated hypotension and bradycardia reflex responses. Our current study has shown that 22% of the rats are non-responsive to EA in the modulation of parasympathoexcitatory response (in the absence of CCK receptor blockade) similar to our sympathoexcitatory reflex response study (Li et al., 2013). These non-responsive rats display responsiveness to EA following blockade of the CCK receptors in the PVN. These data imply that endogenous CCK in the PVN interferes with the actions of EA during the Bezold-Jarisch responses in this region of the hypothalamus.

Restraint stress elicits a variety of behavioral and physiological stress-related responses, including neuroendocrine and cardiovascular responses, the latter being characterized by a sustained increase in blood pressure and heart rate, which last through the restraint period (Lawler et al., 1975; Irvine et al., 1997; McDougall et al., 2005; Tavares and Correa, 2006; Crestani et al., 2010). During the pre-experimental period, animals usually are stimulated with the unavoidable restraint stress. It is conceivable that the pre-experimental restraint stress-related responses interfere with the cardiovascular reflex responses in the absence and presence of EA treatment in the non-responsive rat. However, our findings do not support this assumption. First, the procedures, such as handling performed during the pre-experimental phase, were identical for each rat in both groups of the rats, the responsive and non-responsive rats to the EA treatment. Second, the PBG-evoked cardiopulmonary reflex responses are similar in both responsive and non-responsive rats. Third, studies have demonstrated that EA effectively reduces cardiovascular responses induced by restraint stress (Yang et al., 2002; Imai et al., 2009). Furthermore, the present results have demonstrated that blockade of the CCK-1 receptors converts rats initially EA-unresponsive into EA-responsive. These results jointly imply that the stress-related responses are unlikely involved in the non-responsiveness to EA in the present study.

## Significance and Perspective

Many features of vasovagal syncope are similar to the activation of cardiopulmonary vagal afferents with PBG lowering blood pressure and heart rate. A cardiopulmonary reflex (Aviado and Guevara, 2001) is, in part, a form of neurogenic syncope that is the most common cause of transient unconsciousness (Wieling et al., 2011). We have shown that EA decreases inhibitory hemodynamic cardiopulmonary responses, in particular bradycardia through a GABA_A_ mechanism in the medullary regions nucleus ambiguus and NTS (Tjen-A-Looi et al., 2012, 2014). The current study emphasizes the importance of opioids in the hypothalamic PVN during the actions of EA on a Bezold-Jarisch reflex response.

Many hypotensive responses, for instance, those occurring during alveolar hemorrhage, hypercapnia, and vasovagal syncope, employ the parasympathetic system (Kaufmann, 1995; Gu and Lee, 2002; Lin et al., 2015). Activation of vagal pulmonary C-fibers induced by hemorrhage, chemical stimuli, such as CO_2_, capsaicin, and PBG, results in a profound reduction of blood pressure and heart rate that likely can be modulated by acupuncture, such as EA (Kaufmann, 1995; Gu and Lee, 2002; Syuu et al., 2003; Tjen-A-Looi et al., 2012, 2013; Lin et al., 2015). Importantly, inhibitory actions of EA do not influence baseline blood pressure and heart rate (Li et al., 1998, 2002, 2004; Tjen-A-Looi et al., 2007), implying that EA does not influence basal tone of autonomic output associated with hemodynamic stability. As such, acupuncture therapy could normalize the autonomic tone and does not alter hemodynamic baselines, features that are consistent with the low incidence of side effects during EA application (Li et al., 2004; Tjen-A-Looi et al., 2004). Patients with recurrent cardiovascular depression may benefit from EA therapy operating without altering baseline blood pressure and heart rate.

In conclusion, application of 30 min of bilateral EA at P5-6 acupoints decreases vagally evoked cardiovascular PVN neuronal activity, vasodepression, and bradycardia through the opioid system.

## Data Availability Statement

The raw data supporting the conclusions of this article will be made available by the authors, without undue reservation.

## Ethics Statement

The animal study was reviewed and approved by the University of California, Irvine IACUC.

## Author Contributions

ST-A-L, A-TN, and YG: design and experiment. ST-A-L and YG: data analyses. ST-A-L: manuscript preparation. L-WF, Z-LG, SM, AN, and YG: manuscript review. All authors contributed to the article and approved the submitted version.

## Conflict of Interest

The authors declare that the research was conducted in the absence of any commercial or financial relationships that could be construed as a potential conflict of interest.

## Publisher’s Note

All claims expressed in this article are solely those of the authors and do not necessarily represent those of their affiliated organizations, or those of the publisher, the editors and the reviewers. Any product that may be evaluated in this article, or claim that may be made by its manufacturer, is not guaranteed or endorsed by the publisher.
